# Genome-wide identification and molecular characterization of *CRK* gene family in cucumber (*Cucumis sativus* L.) under cold stress and *sclerotium rolfsii* infection

**DOI:** 10.1186/s12864-023-09319-z

**Published:** 2023-04-26

**Authors:** Satyabrata Nanda, Priyadarshini Rout, Ikram Ullah, Swapna Rani Nag, Velagala Veerraghava Reddy, Gagan Kumar, Ritesh Kumar, Shuilian He, Hongzhi Wu

**Affiliations:** 1 MS Swaminathan School of Agriculture, Centurion University of Technology and Management, Paralakhemundi, 761211 India; 2 College of Landscape and Horticulture, Yunnan Agricultural University, Kunming, 650201 China; 3 Krishi Vigyan Kendra, Narkatiaganj, Dr. Rajendra Prasad Central Agricultural University, Pusa Samastipur, Bihar, 848125 India

**Keywords:** Cucumber, Genome-wide identification, Cold stress, Pathogen infection, *Sclerotium rolfsii*

## Abstract

**Background:**

The plant cysteine-rich receptor-like kinases (*CRK*s) are a large family having multiple roles, including defense responses under both biotic and abiotic stress. However, the *CRK* family in cucumbers (*Cucumis sativus* L.) has been explored to a limited extent. In this study, a genome-wide characterization of the *CRK* family has been performed to investigate the structural and functional attributes of the cucumber *CRK*s under cold and fungal pathogen stress.

**Results:**

A total of 15 *C. sativus CRK*s (*CsCRK*s) have been characterized in the cucumber genome. Chromosome mapping of the *CsCRK*s revealed that 15 genes are distributed in cucumber chromosomes. Additionally, the gene duplication analysis of the CsCRKs yielded information on their divergence and expansion in cucumbers. Phylogenetic analysis divided the CsCRKs into two clades along with other plant CRKs. Functional predictions of the *CsCRK*s suggested their role in signaling and defense response in cucumbers. The expression analysis of the *CsCRK*s by using transcriptome data and via qRT-PCR indicated their involvement in both biotic and abiotic stress responses. Under the cucumber neck rot pathogen, *Sclerotium rolfsii* infection, multiple *CsCRK*s exhibited induced expressions at early, late, and both stages. Finally, the protein interaction network prediction results identified some key possible interacting partners of the *CsCRK*s in regulating cucumber physiological processes.

**Conclusions:**

The results of this study identified and characterized the *CRK* gene family in cucumbers. Functional predictions and validation via expression analysis confirmed the involvement of the *CsCRK*s in cucumber defense response, especially against *S. rolfsii*. Moreover, current findings provide better insights into the cucumber CRKs and their involvement in defense responses.

**Supplementary Information:**

The online version contains supplementary material available at 10.1186/s12864-023-09319-z.

## Background

Plant resilience and productivity are challenged by several biotic stresses in its natural environment. In these stresses, pathogen infection is a major threat to plants. Plants have developed specialized strategies, such as innate immunity and systemic acquired resistance to tackle these adversities. These defense responses are conferred by several classes of plant proteins either by recognizing the pathogens or pathogen-associated signatures or by orchestrating the downstream signaling cascades [1]. The receptor-like kinases (RLKs) are one such superfamily of plant proteins that functions in the perception of different stimuli and signals [2]. The cysteine-rich receptor-like kinases (CRKs) family belongs to the RLK superfamily and has been well-studied in plants. The CRKs possess three distinct domains, including the extracellular domain, transmembrane domain, and intracellular kinase domain [3]. In plants, the signals are perceived by the extracellular domain, which subsequently passes through the transmembrane domain, and lastly transduced by the intracellular kinase domain [4]. In addition, the CRKs contain the Domain of Unknown Function 26 (DUF26) domains and thus, also known as DUF26-RLKs with a signature C-X_8_-C-X_2_-C motif [5]. Interestingly, the DUF26 has not been attributed with any confirmed functions, however, predicted to be playing a role in regulating the three-dimensional structure of the CRKs and in the protein-protein/DNA interactions [4].

The *CRK*s are important players in regulating defense responses and plant-pest interactions. Their roles have been studied in several model and non-model plants in conferring resistance against phytopathogens. For instance, the overexpression of *AtCRK5*, *AtCRK6*, *AtCRK36*, and *AtCRK45* protected the Arabidopsis plants against *Pseudomonas syringae* infection through the induced expression of defense-responsive genes and activation of reactive oxygen species (ROS) signaling [6, 7]. In addition, *CRK*s can regulate plant defense responses by modulating important signaling cascades, such as phytohormonal pathways, including salicylic acid (SA) and jasmonic acid (JA), and programmed cell death [8–10]. Recently, the *CRK*s in chili pepper were found to be involved in chili-*Colletotrichum truncatum* interactions. In addition, the *CRK*s have been reported to exhibit differential expressions in response to several abiotic stresses, including cold stress. For instance, multiple *GhCRK*s were induced under cold stress in cotton [11]. Due to their important roles, the *CRK* family has been characterized in several plants, including Arabidopsis, tomato, common bean, cotton, and chili pepper [3, 4, 12, 13]. However, no substantial information is available on the *CRK* gene family in the important vegetable cucumber. Therefore, it is important to identify and characterize the *CRK*s in cucumbers and investigate their involvement in cucumber defense response.

Cucumber (*Cucumis sativus* L.) is an important vegetable with food, medicinal, and economic significance. However, the production of cucumber is severely challenged by several pathogens, such as powdery mildew, downy mildew, anthracnose, collar rot, and so on. Among them, cucumber collar rot caused by the fungal pathogen *Sclerotium rolfsii* is a serious disease that causes wilting and rotting of stems, occasionally fruits, resulting in severe crop damage [14, 15]. At present, early detection and application of fungicides are the best methods of controlling *S. rolfsii* in cucumbers. However, understanding the molecular mechanisms of cucumber- *S. rolfsii* interactions will add new insights into the pathogenicity of *S. rolfsii* and cucumber defense responses. As *CRK*s regulate the defense responses in several plants, exploring the involvement of *CRK*s in cucumber-*S. rolfsii* interactions will be beneficial. Moreover, the availability of the cucumber genome sequence in the public domain facilitates the genome-wide identification of gene families and their subsequent functional characterizations. Several gene families have been recently identified in the cucumber genome, including the Lectin Receptor-Like Kinase (LecRLK), CLAVATA3/Embryo surrounding region-related (CLE), Teosinte branched1/Cycloidea/Proliferating cell factor (TCP), trehalose-6-phosphate synthase (TPS), WUSCHEL-related homeobox (WOX), and CC-NBS-LRR(CNL) [16–20]. In this study, 15 *C. sativus CRK*s (named as *CsCRK*s) have been identified in the cucumber genome. The identified *CsCRK*s were structurally characterized by stringent *in silico* analysis, including protein properties predictions, motif and domain analysis, phylogenetic classification, chromosomal mapping, gene structure organization, gene ontology (GO) analysis, *cis*-regulatory element predictions, gene duplication, and synteny and colinearity analysis. In addition, the expression of the *CsCRK*s was verified under cold stress and powdery mildew infection by using the transcriptome data. Furthermore, the involvement of *CsCRK*s was investigated during cucumber-*S. rolfsii* interactions through real-time quantitative polymerase chain reaction (RT-qPCR).

## Results

### Identification and characterization of *CsCRK*s in cucumber

**Fig. 1:**
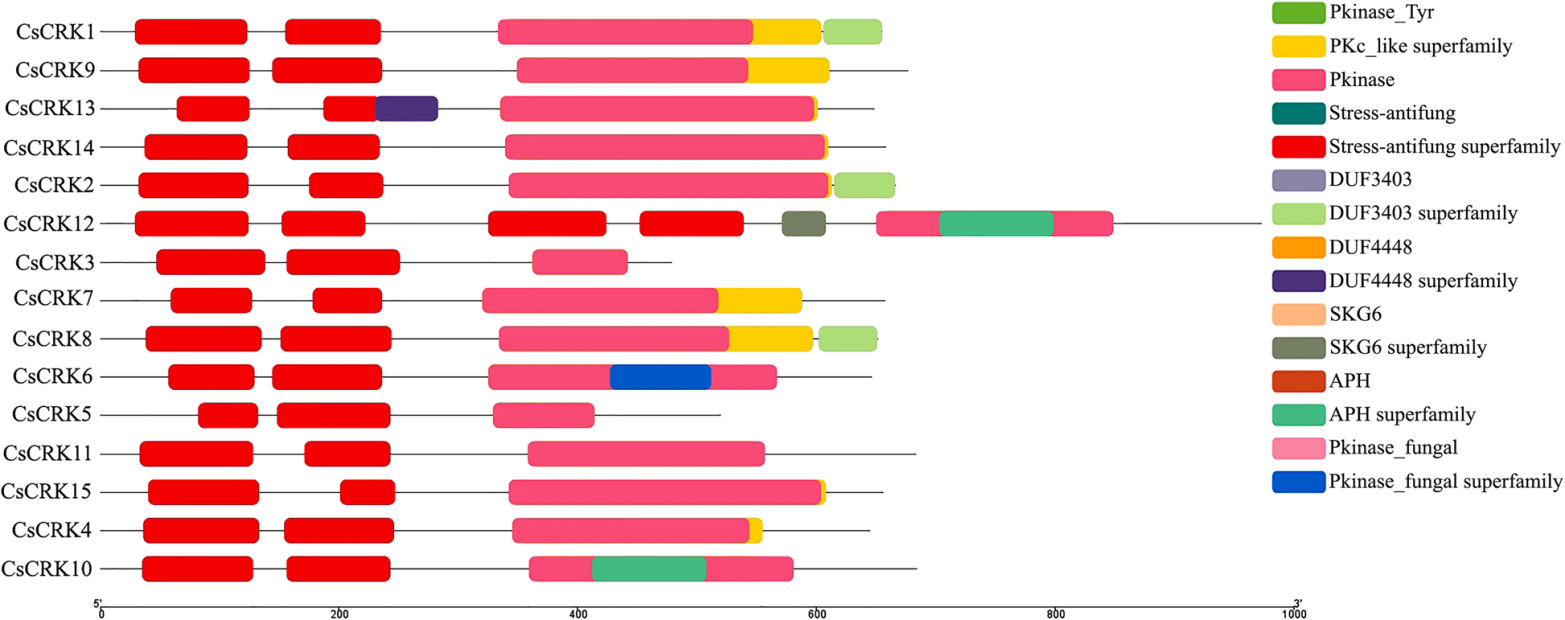
The presence of different conserved domains in CsCRK sequences

A set of stringent bioinformatics analyses resulted in the identification of 15 *CRK*s in the cucumber genome and they were named *CsCRK1* to *CsCRK15* as per their distribution in the *C. sativus* chromosomes. All these identified genes contained the signature Stress-antifung (PF01657) and Pkinase (PF00069) Pfam domains, along with other conserved domains as verified by using the Conserved Domain Database (CDD) and Simple Modular Architecture Research Tool (SMART) tools (Fig. 1). The peptide properties of the CsCRKs were predicted by using the Protparam program. The results revealed that the molecular weight of the CsCRKs varied in the range of 52.96 KDa (CsCRK15) to 108.31 (CsCRK9) (Table 1). Likewise, the predicted iso- electric points (pI) range varied from 5.92 (CsCRK15) to 9.10 (CsCRK2). All the identified CsCRKs exhibited a negative GRAVY score suggesting their hydrophilic nature.

**Fig. 2:**
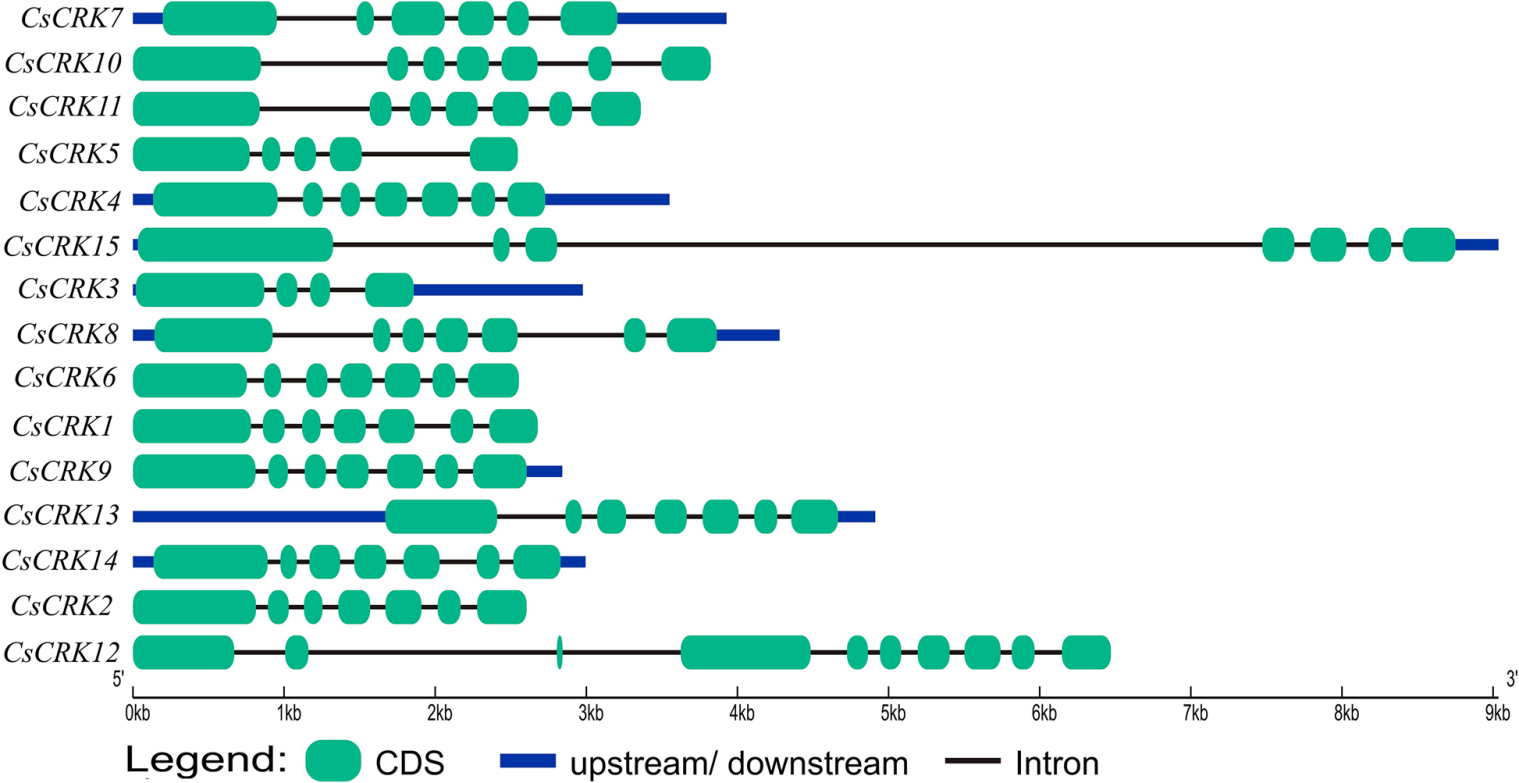
Identification of the exon-intron assembly in *CsCRK*s. Denotations for the cyan boxes and different colored lines are mentioned at the bottom

**Fig. 3:**
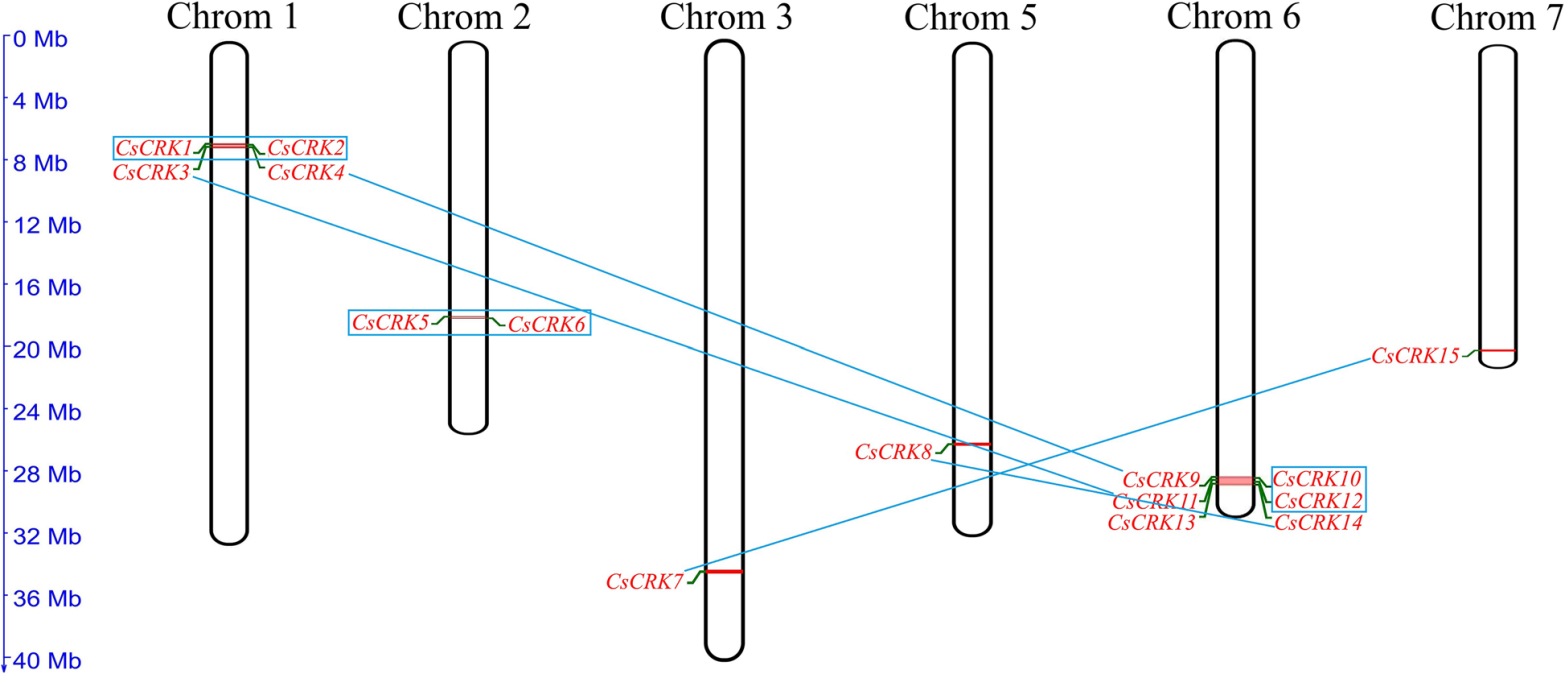
Distribution of *CsCRK*s on different cucumber chromosomes. The side ruler indicates the individual chromosome sizes. Genes originated from tandem and segmental duplication are marked by boxes and straight lines, respectively

The exon-intron organization of the identified 15 *CsCRK*s was analyzed by employing the GSDS program. The number of exons was found to be in the range of 10 (*CsCRK12*) to 4 (*CsCRK3*) (Fig. 2). Further, all 15 *CsCRKs* were mapped onto the cucumber chromosomes. The chromosomal distribution of the *CsCRKs* was not equal. For example, six *CRKs* were mapped onto chromosome 6, whereas chromosomes 3, 5, and 7 contained one each (Fig. 3). On the other hand, the subcellular localization prediction results indicated that most of the CsCRKs might localize in the nucleus or on the plasma membrane (Table 1).

### Motif and phylogenetic analysis of *CsCRK*s

**Fig. 4:**
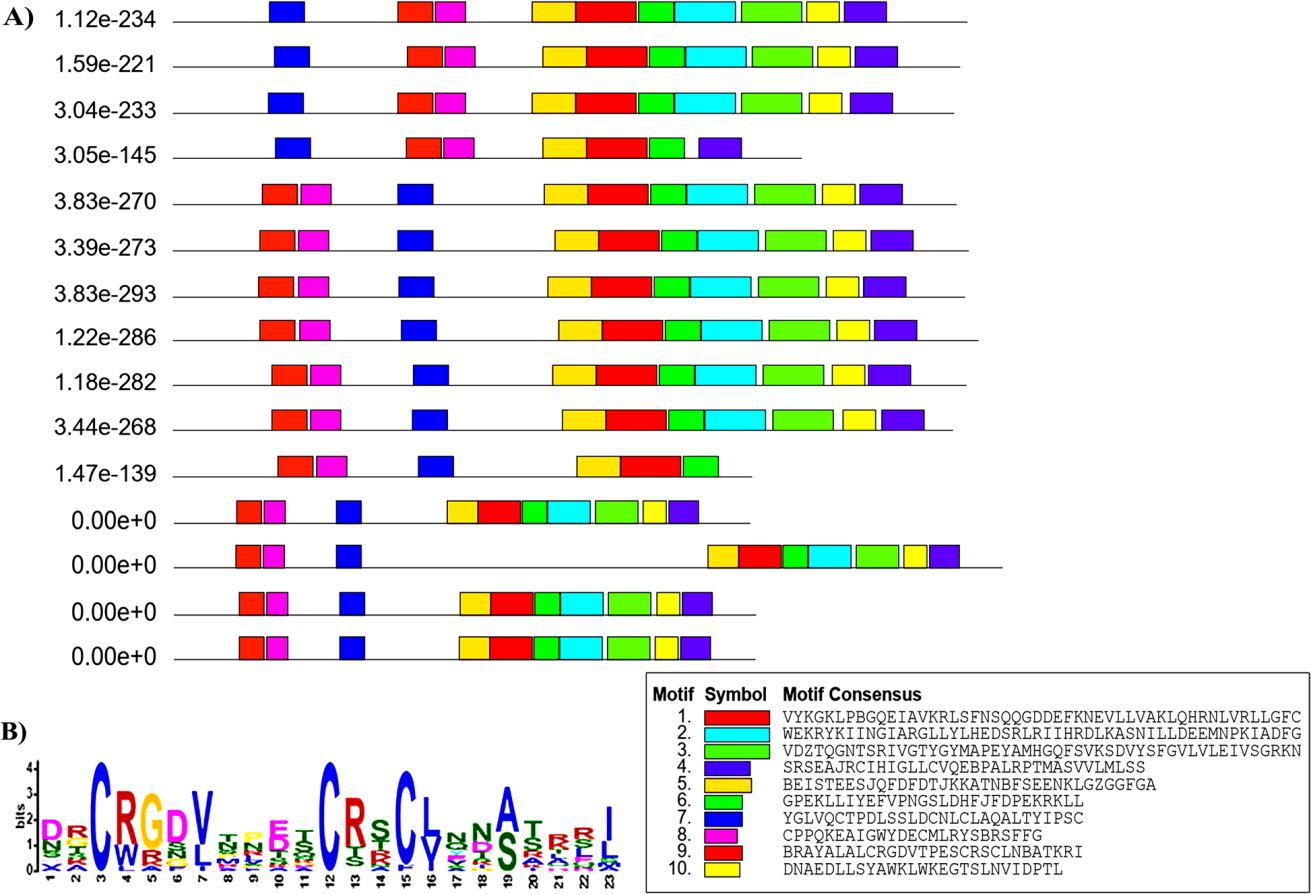
** A** Distribution and analysis of motifs in the CsCRK sequences. The solid line indicates the individual protein sequence length, whereas the colored boxes are the different identified motifs. The sequence of each motif is provided at the bottom of the picture. **B** The evolutionary conserved C-X_8_-C-X_2_-C motif obtained from MEME analysis

**Fig. 5:**
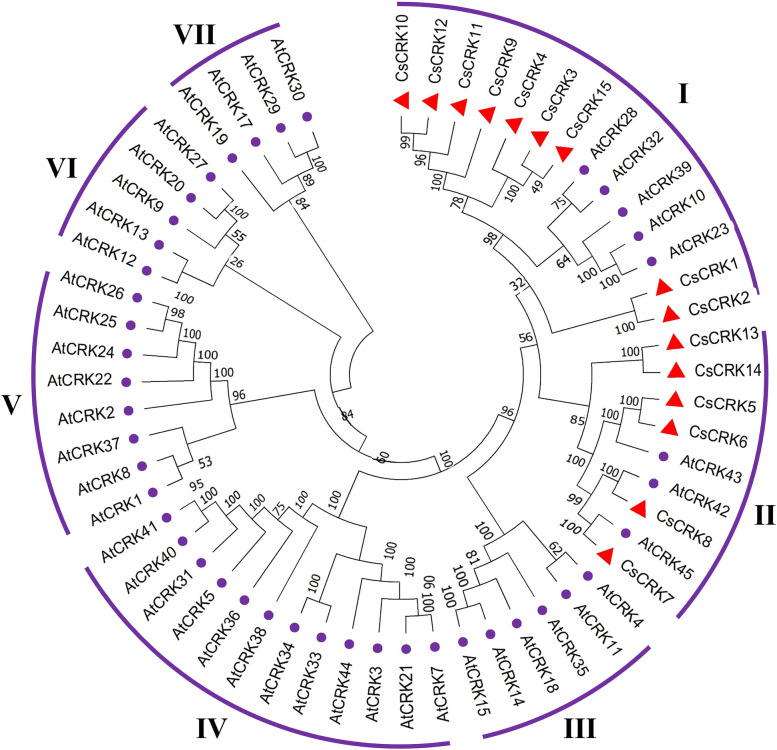
Phylogenetic analysis of the CsCRKs among other plant CRKs through the neighbor-joining method with 1000 bootstraps by using MEGA v11. Individual sub-groups on the tree have been marked with the roman numeric. The red triangles denote the CRKs in cucumber, while the purple dots denote the Arabidopsis CRKs

A MEME-based de novo motif analysis strategy was employed to elucidate the conserved motifs in the CsCRKs (Fig. 4). All 15 CsCRKs were found to contain the highly conserved C-X_8_-C-X_2_-C consensus and the DUF26 signature domain. In addition, a phylogenetic analysis was conducted by including the CsCRKs and AtCRKs to check their ancestral relationship. The phylogenetic tree divided all the CRKs into seven groups (I-VII). The CsCRKs were distributed in two of the seven groups on the tree; group I had 9 CsCRKs, while 6 were placed in group II (Fig. 5).

**Table 1:** The predicted physicochemical properties of *CsCRK*s in cucumbers

Name	Transcript ID	Chrom no.	Location (Start-End)	Strand	Exons	Amino acids	MW (KDa)	pI	GRAVY Score	Localization
*CsCRK1*	CsaV3_3G042660.1	3	34,624,574–34,628,501	+ ve	7	657	72.94	7.46	-0.090	Plasma membrane
*CsCRK 2*	CsaV3_5G031370.1	5	25,639,895–25,644,173	+ ve	7	651	72.16	9.10	-0.104	Plasma membrane
*CsCRK3*	CsaV3_2G024830.1	2	17,186,289–17,188,842	– ve	4	646	71.27	8.22	-0.140	Nucleus
*CsCRK4*	CsaV3_1G011140.1	1	6,892,215–6,894,893	+ ve	7	655	73.65	8.31	-0.164	Nucleus
*CsCRK5*	CsaV3_6G049310.1	6	28,824,485–28,827,325	+ ve	5	677	75.57	6.55	-0.165	Nucleus
*CsCRK6*	CsaV3_6G049370.1	6	28,857,428–28,862,339	– ve	7	648	72.09	5.96	-0.129	Nucleus
*CsCRK7*	CsaV3_6G049380.1	6	28,863,435–28,866,429	– ve	6	658	73.41	6.27	-0.190	Endoplasmic-Reticulum
*CsCRK8*	CsaV3_1G011160.1	1	6,903,507–6,906,111	+ ve	7	666	74.53	6.80	-0.111	Plasma membrane
*CsCRK9*	CsaV3_6G049360.1	6	28,849,942–28,856,412	+ ve	7	973	108.31	7.98	-0.161	Nucleus
*CsCRK10*	CsaV3_6G049320.1	6	28,828,428–28,832,251	+ ve	7	684	75.47	8.34	-0.185	Nucleus
*CsCRK11*	CsaV3_6G049350.1	6	28,841,610–28,844,970	+ ve	7	683	76.63	6.34	-0.181	Plasma membrane
*CsCRK12*	CsaV3_2G024820.1	2	17,182,714–17,185,260	– ve	10	520	57.87	8.71	-0.249	Nucleus
*CsCRK13*	CsaV3_1G011280.1	1	6,962,082–6,965,632	+ ve	7	645	72.26	7.21	-0.202	Nucleus
*CsCRK14*	CsaV3_7G033220.1	7	20,963,946–20,973,125	– ve	7	656	73.98	7.51	-0.200	Nucleus
*CsCRK15*	CsaV3_1G011200.1	1	6,917,825–6,920,801	+ ve	7	479	52.96	5.92	-0.003	Nucleus

*CDS* coding DNA sequence, *MW* Molecular weight, *pI* Isoelectric point, *GRAVY* Grand average of hydropathicity

### Gene duplication, synteny, colinearity, *cis*-regulatory element, and GO analysis of *CsCRK*s

**Fig. 6:**
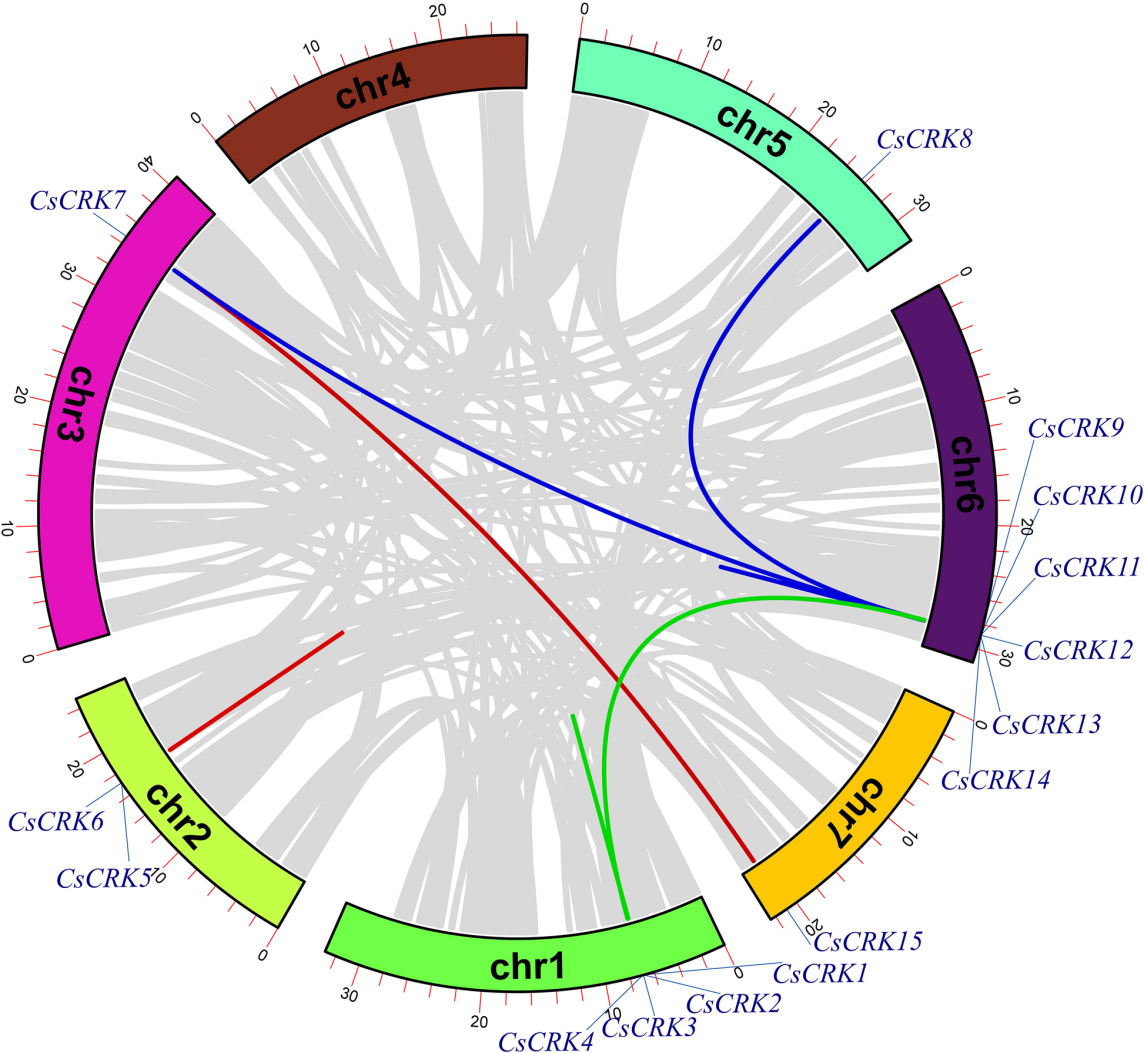
Synteny analysis and inter-chromosomal relationships of the *CsCRK*s. The colored lines represent the duplicated *CsCRK* pairs in the cucumber genome. Chromosomes are named in different colored boxes

**Fig. 7:**
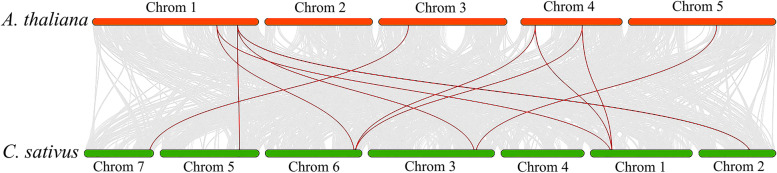
Colinearity analysis of *CRK* genes in cucumber with the Arabidopsis *CRKs*. The collinear blocks are indicated by the background gray lines within cucumber and Arabidopsis genomes, while the syntenic *CRK* gene pairs are denoted by the red lines. Each chromosome number is shown above the chromosomes of the respective species

The duplication events of the identified *CsCRK*s were predicted by synteny analysis and by estimating the Ka/Ks ratios. A pairwise comparison among all 15 *CsCRK*s resulted in a range of 0.36 to 0.63 Ka/Ks ratio with six possible duplicated gene pairs, including *CsCRK2-CsCRK1, CsCRK4-CsCRK9, CsCRK5-CsCRK6, CsCRK10-CsCRK12, CsCRK11-CsCRK3*, and *CsCRK14-CsCRK8* (Fig. 6, Table S2), indicating a negative selection in the *CsCRK* gene family. In addition, to get better insights into the evolution of *CRK* genes in cucumber, the colinearity analysis between the *CsCRKs* and *AtCRKs* was analyzed (Fig. 7). The results showed that 11 *CsCRK*s showed colinearity with *AtCRK*s indicating these could be the ortholog pairs. Similarly, the *cis*-regulatory elements analysis of the *CsCRK*s showed the presence of several classes of *cis*-elements, including the phytohormone-associated elements such as salicylic acid (SA)-responsive element (TCA-element), jasmonic acid (JA)-and elicitor-responsive element (JERE), abscisic acid (ABA)-responsive element (ABRE), and ethylene (ET)-responsive element (ERE) (Fig. S1).

**Fig. 8:**
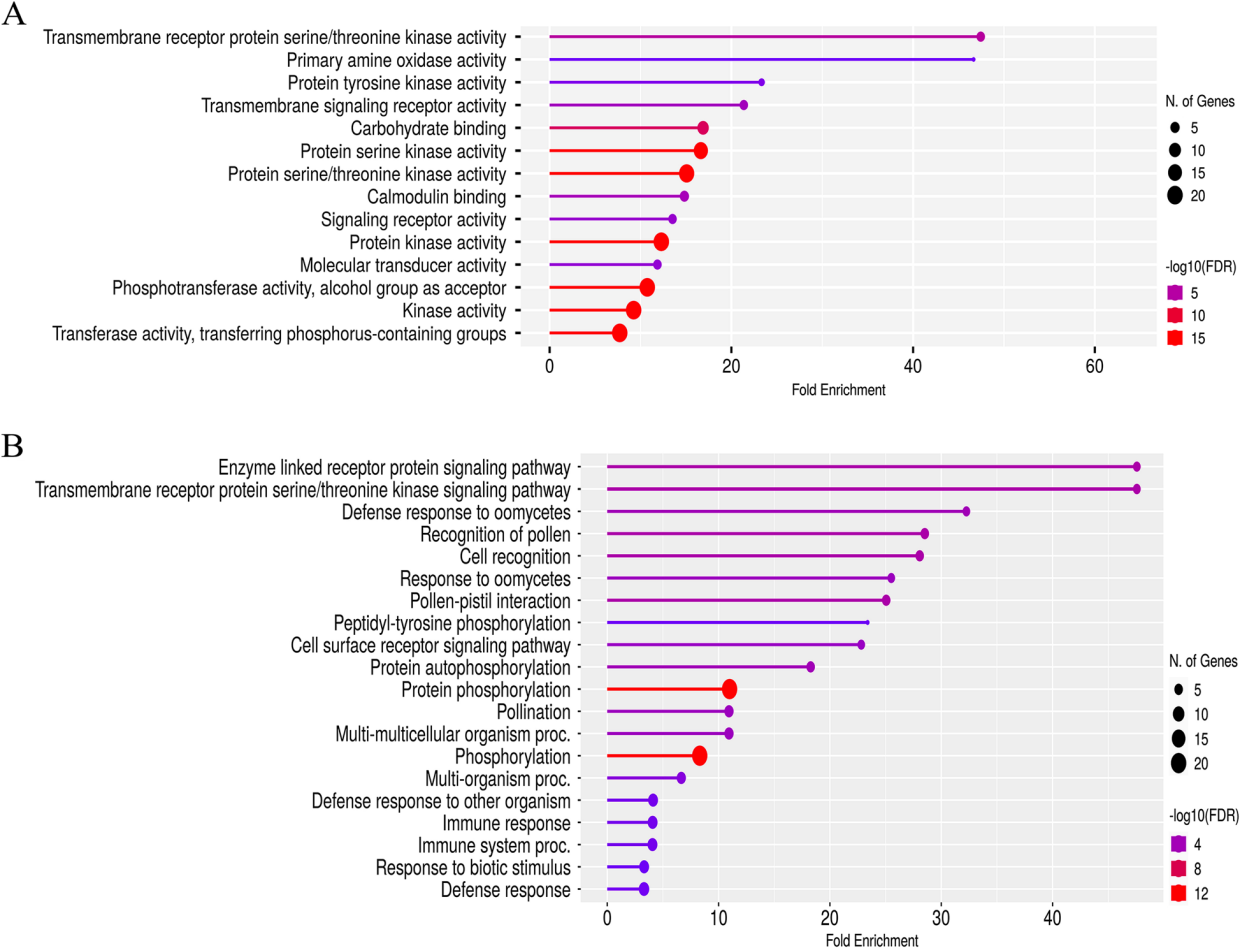
Gene ontology (GO) analysis of the identified *CsCRK*s. **A** Prediction of the most enriched molecular functions. **B** Prediction of the most enriched biological processes. Here, the N. of Genes legend represents the number of *CsCRK*s and the relative circle size is indicative of the relative number of genes

The GO analysis of the *CsCRKs* revealed their putative molecular and biological functions. For instance, the most enriched molecular function of the *CsCRK*s was found to be protein kinase activity, while the most enriched biological functions were signaling pathway, defense response, and protein phosphorylation (Fig. 8).

### Expressions analysis of *CsCRK*s by using transcriptome data

**Fig. 9:**
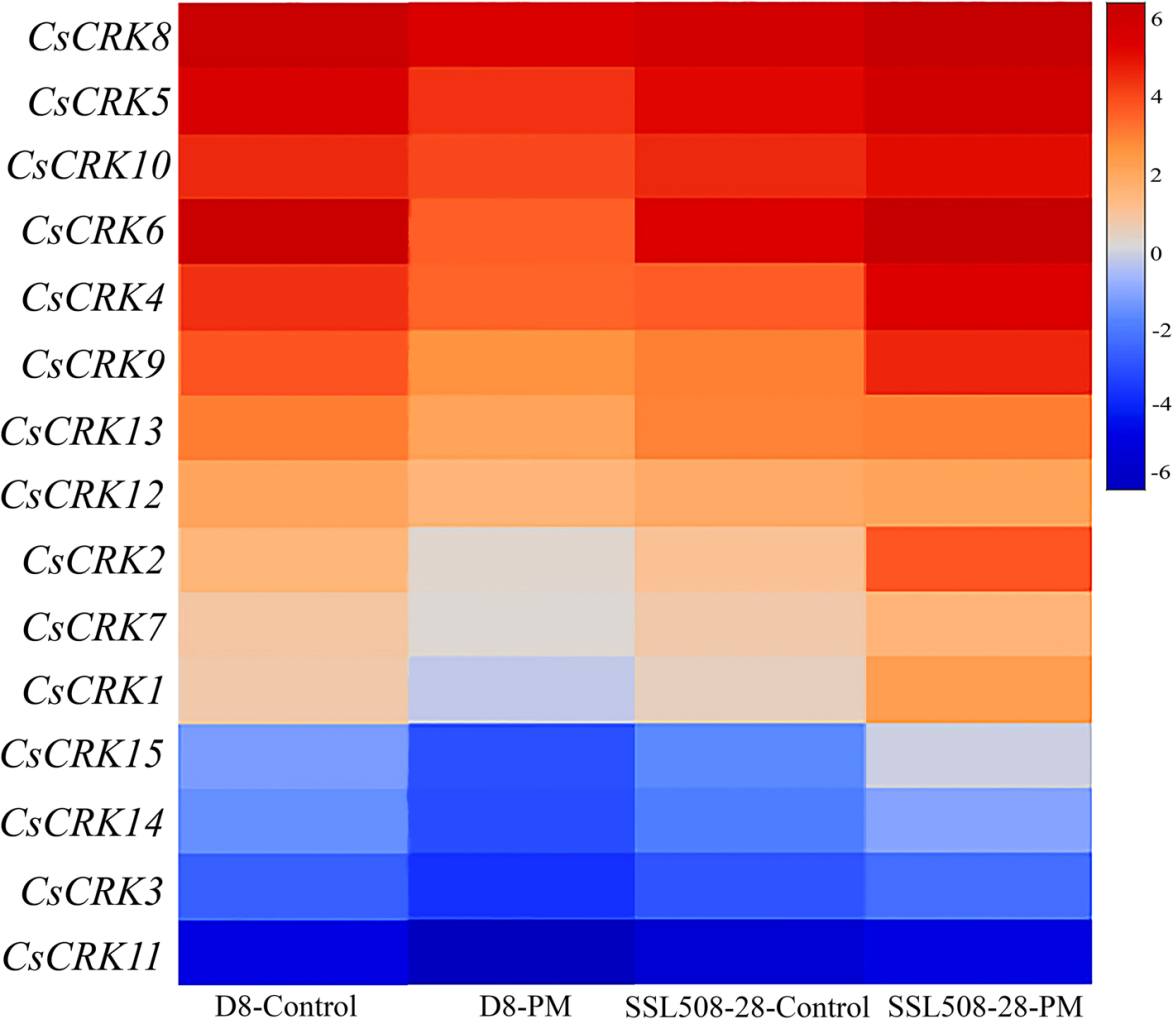
Heat map showing the expression profiles of the *CsCRK*s under powdery mildew (PM) stress in cucumber at 2 DPI. CT: control; PM: inoculated with PM pathogen; D8: susceptible cucumber variety; SSL508-28: the segment substitution PM-resistant cucumber line. The red color shows the upregulation and the blue color shows the downregulation of the gene expressions

**Fig. 10:**
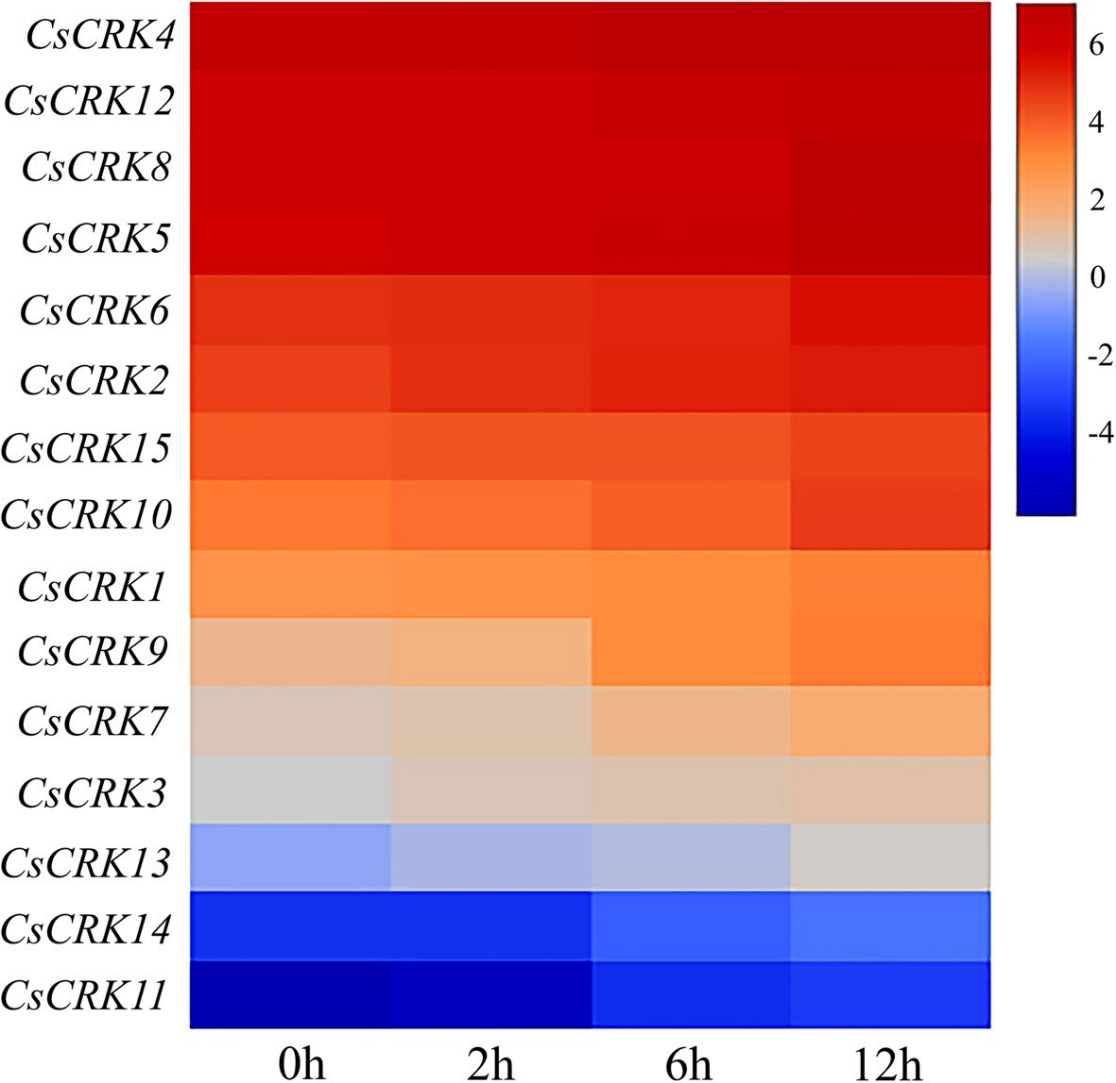
Heat map showing the expression profiles of the *CsCRK*s under cold stress in cucumber. The red color shows the upregulation and the blue color shows the downregulation of the gene expressions

The presence of several stress-responsive regulatory elements in the promoters of the *CsCRK*s suggested their involvement in cucumber defense response. To further verify that, we have used the transcriptome data available on the CuGenDB website. From the transcriptome data, the differential expressions of the *CsCRK*s were deduced in response to both biotic (powdery mildew, PM) and abiotic (cold) stresses. In response to PM infection, the expression of the *CsCRK*s was checked in the transcriptome of two contrasting varieties after 2 days post inoculation (DPI); D8 susceptible and SSL508-28 resistant to PM, respectively. The results revealed that multiple *CsCRK*s were involved in response to PM infection in both varieties. However, the PM infection caused possible downregulation of the *CsCRK*s in the susceptible D8 variety as compared to the uninfected plant (control). Conversely, in the resistant SSL508-28 variety, the PM infection upregulated several *CsCRK*s as compared to the control (Fig. 9). Similarly, under cold stress, the *CsCRK*s displayed a differential temporal expression pattern. Multiple *CsCRK*s, including *CsCRK4, CsCRK12, CsCRK8, CsCRK5, CsCRK6, CsCRK2, CsCRK15*, and *CsCRK10* showed an induced expression in response to the cold stress (Fig. 10). In addition, their expression levels were increased with time up to the 12 h time point as compared to 0 h. The induced expression of *CsCRK4, CsCRK12, CsCRK8*, and *CsCRK5* remained almost constant, while the expression of *CsCRK6, CsCRK2, CsCRK15*, and *CsCRK10* increased with time as compared to the other *CsCRK*s. Overall, these results suggest that *CsCRK*s could be involved in responding to both biotic and abiotic stresses in cucumbers.

### Expressions analysis of *CsCRK*s in response to *S. rolfsii*

**Fig. 11:**
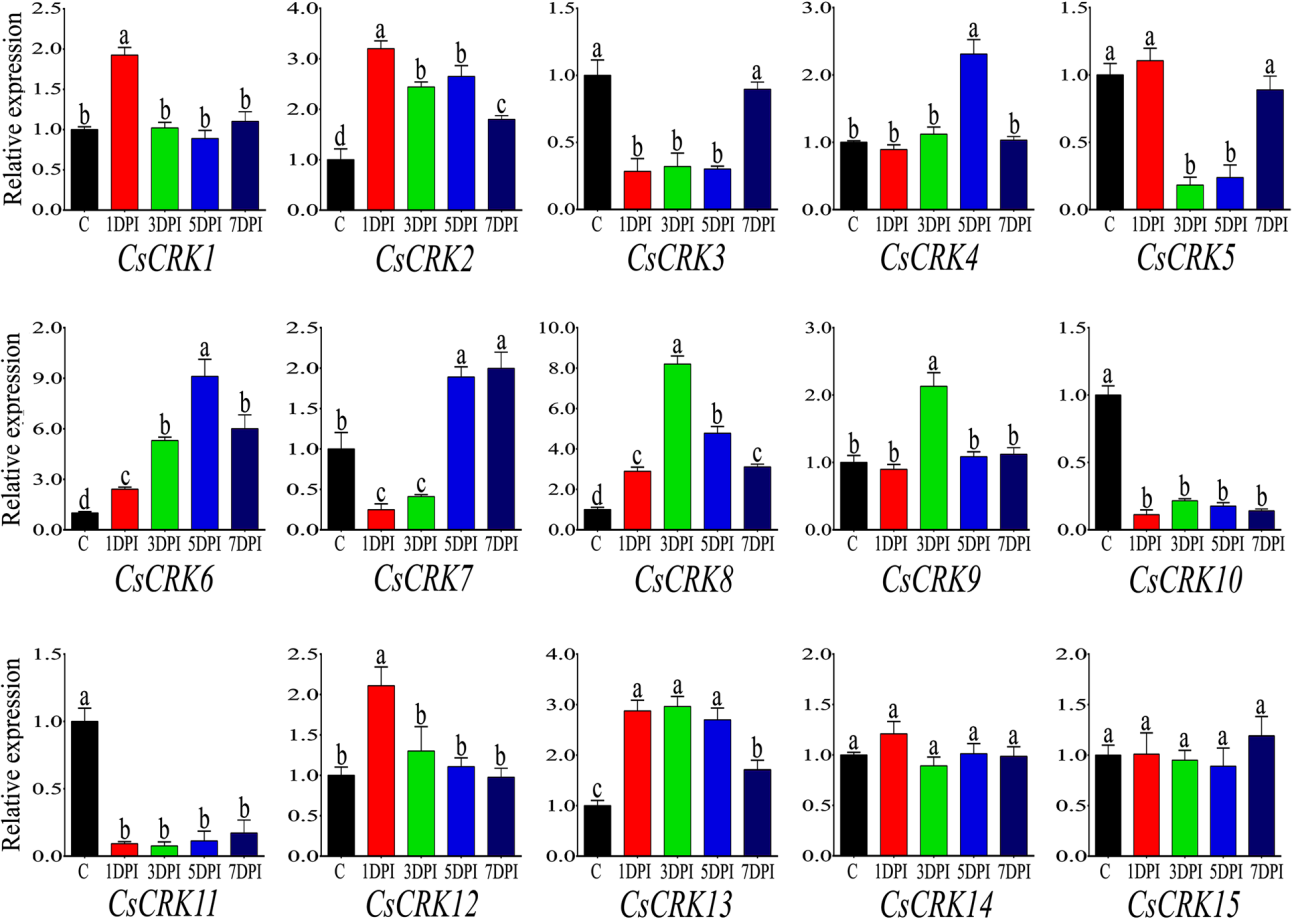
The expression profiles of all 15 *CsCRK*s under *S. rolfsii* infection. The relative expression levels (fold changes) of the *CsCRK*s are indicated at the Y-axis. The lowercase alphabets (a-e) denote the statistical significance of the expression at *P* < 0.05. Data are represented as mean ± SE. DPI: days-post inoculation

The qRT-PCR-mediated expression analysis results revealed that multiple *CsCRK*s exhibited differential expression patterns in response to the *S. rolfsii* infection. Out of the 15 *CsCRK*s, 9 (*CsCRK1, CsCRK2, CsCRK4, CsCRK5, CsCRK6, CsCRK7, CsCRK8, CsCRK9, CsCRK12*, and *CsCRK13*) of them were upregulated either at 1, 3, 5, or 7 DPI (Fig. 11). Out of them, *CsCRK1, CsCRK9*, and *CsCRK12* exhibited an early induced expression (on 1 or 3 DPI), whereas *CsCRK3*, *CsCRK4*, and *CsCRK7* displayed a late induced expression (on 5 or 7 DPI). *CsCRK3, CsCRK5, CsCRK6, CsCRK8*, and *CsCRK13* were found to show both early and late induced expression in response to *S. rolfsii* infection. On the other hand, *CsCRK10* and *CsCRK11* exhibited a downregulated expression for all 4-time points. Interestingly, the expressions of *CsCRK3* and *CsCRK7* were first downregulated and then upregulated in response to the *S. rolfsii* infection. *CsCRK14* and *CsCRK15* exhibited no significant changes in their expressions under the *S. rolfsii* infection as compared to the control.

### Predicted protein-protein interaction network of *CsCRK*s

**Fig. 12:**
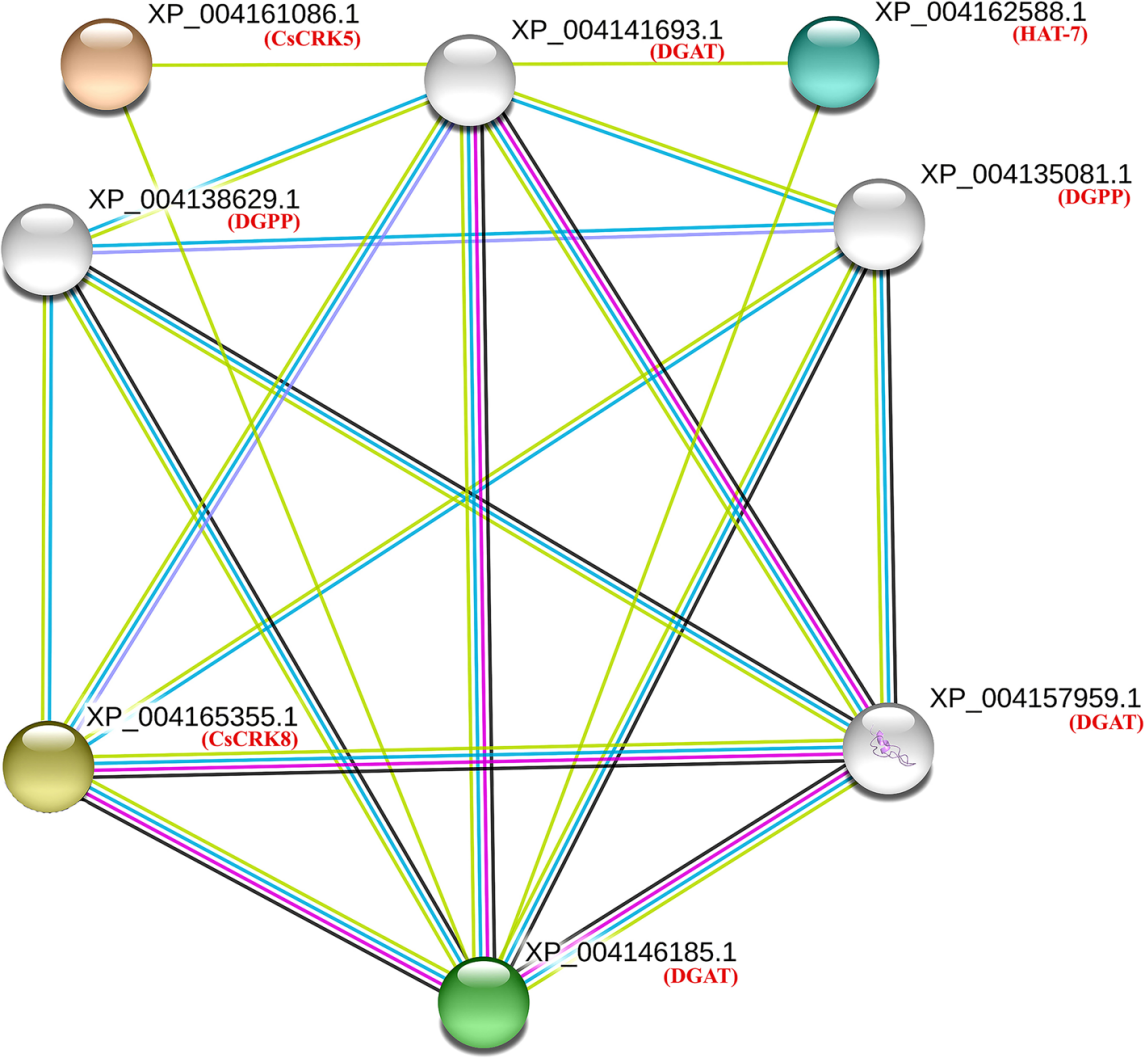
Prediction of the protein interaction network by using the STRING tool. The different colored nodes represent the interacting proteins, and the edges represent protein-protein associations. The black line indicates the coexpression evidence, the purple line indicates the experimental evidence, the light blue line indicates the database evidence, and the green line indicates the neighborhood evidence, respectively

The CRKs are a vital class of protein having several interacting partners in the cell. Here, we tried to predict the protein interaction network of the CsCRKs in cucumber by using the STRING program. The interaction network was constructed among 8 proteins, having 2 CsCRKs (CsCRK5 and CsCRK8) and 6 interacting proteins, including 3 diacylglycerol acyltransferases (DGAT; XP_004141693.1, XP_004157959.1, and XP_004146185.1), 2 diacylglycerol pyrophosphates (DGPP; XP_004138629.1 and XP_004135081.1), and 1 homeobox-leucine zipper protein HAT7-like (HAT-7; XP_0016258.1) (Fig. 12).

## Discussion

In plants, the *CRK*s have been reported to be involved in defense responses against multiple stresses, including pathogen infections [21–23]. In cucumber, a dominantly inherited major quantitative trait locus (QTL), *Pm1.1* was identified to contribute powdery mildew resistance, which contained two *CRK* genes in tandem [24]. Although the cucumber genome has been sequenced, the number of *CRK* genes in the cucumber genome is unclear. By utilizing the available genome sequences and by stringent bioinformatics analysis several plant gene families, including the *CRK*s have been characterized [3, 4, 12, 13, 25]. In this study, a total of 15 cucumber *CRK*s (*CsCRK*s) have been identified and their in silico characterization confirmed the presence of conserved signature domains and motifs. The phylogenetic analysis divided the *CsCRK*s along with other plant *CRK*s into 7 groups. However, the *CsCRK*s were distributed in only 2 out of the 7 groups. A similar grouping of the *CRK*s was reported in some previous studies where the identified *CRK*s were clustered in only 2 sub-groups in the phylogenetic tree [3]. The possible reason for this could be the divergence in *CRK* evolution in cucumbers as compared to other plants.

The GO analysis results suggested that the *CsCRK*s are mostly involved in the signaling pathways and defense response in cucumbers. Earlier reports have confirmed the role of CRKs in stimulus perception, downstream signaling, and modulation of defense responses in plants [26, 27]. Additionally, protein phosphorylation has been identified as one of the most enriched molecular functions of the *CsCRK*s in GO analysis. Protein phosphorylation is a key event in the signaling cascades and is crucial in regulating plant defense [28]. In Arabidopsis, AtCRK36 enhances the flg22-triggered phosphorylation of *Botrytis-induced kinase1 (BIK1)* and modulates the Arabidopsis defense [14]. The localization prediction results revealed that the *CsCRK*s either localize in the nucleus or on the plasma membrane. Overexpression of the membrane-localized Arabidopsis *CRK*, *AtCRK5* regulated the abscisic acid sensitivity conferring drought tolerance [29]. Overall, these findings further strengthen the involvement of the *CsCRK*s in cucumber defense responses.

The expression analysis results of *CsCRK*s by using the transcriptome data suggested that *CsCRK*s respond to both abiotic and biotic stressors in cucumbers. The transcription of multiple *CsCRK*s got upregulated temporally under cold stress. In cotton, multiple *CRK*s exhibited induced expressions in response to cold stress [27]. On the other hand, under PM infection, interesting *CsCRK* expression patterns were observed in two contrasting cucumber varieties. In the PM-resistant variety, the *CsCRK*s exhibited elevated expressions in response to PM infection as compared to the control, whereas in the susceptible varieties, the *CsCRK*s were downregulated under PM infection. The contrasting expression patterns of the *CsCRK*s in the resistant and susceptible cucumber varieties suggest that the expression of *CsCRK*s can be linked to cucumber resistance response. In addition, the *CsCRK*s could be the positive regulator of cucumber defense, and by downregulating the *CsCRK* expressions in a susceptible variety, the PM pathogen might be winning the arms race. In Arabidopsis, *AtCRK45* was reported to be a positive regulator of the defense responses against *P. syringae* infection [6]. Similarly, the wheat *CRK*, *TaCRK2*, positively regulates wheat resistance against leaf rust disease [30]. Here, the qRT-PCR results confirmed that 9 out of 15 identified *CsCRKs* were induced in response to the *S. rolfsii* inoculation. The expression of *CsCRK*s was observed in three different trends; early expression, late expression, and both early and late expressions. Similar trends of expressions of *CRK*s have been reported in cotton and chili plants in response to fungal infections [3, 4]. Taken together, the results indicate that the *CsCRK*s are involved in the cucumber defense responses, both against abiotic and biotic stresses. Overall, their expression dynamics can have a variety of specific and time-specific trends in response to a particular stress.

To further understand the function of the *CsCRK*s, we predicted the protein interaction network. The results suggested that the CsCRKs might interact with different proteins in cucumber in regulating various physiological processes. We identified 6 interacting proteins, including 3 diacylglycerol acyltransferases, 2 diacylglycerol pyrophosphates, and 1 homeobox-leucine zipper protein HAT7-like to interact with CsCRKs. The DGAT is the rate-limiting enzyme in the synthesis of triacylglycerol (TAG) [31], while TAG has been reported to be an alternative energy source in the cell and could be a player in stress response [32]. Similarly, DGPP is a key phospholipid which is virtually remains absent in normal cells. However, up to the onset of stress signals, including both biotic and abiotic stresses, its concentration gets higher suggesting its involvement in stress signaling [33, 34]. On the other hand, HAT7 has been reported to be involved in modulating the growth and development of plants [35]. Thus, the interactions of the CsCRKs among the aforementioned proteins suggest that the CsCRKs might be involved in regulating the defense response and plant development in cucumbers. However, in-depth research and functional validation will further confirm the predicted interactions of the CsCRKs among other proteins.

## Conclusion

The study reports the identification and characterization of 15 *CsCRK*s in the cucumber genome. The extensive *in silico* characterization revealed their canonical conserved motifs, domains, gene structures, and other physio-chemical properties. Additionally, analysis of the promoter sequences of the *CsCRK*s suggested their putative functions as per the *cis*-regulatory elements present in them. The functional predictions were further strengthened by the GO analysis which suggested that *CsCRK*s could mostly be involved in signaling and defense responses with major molecular functions such as protein phosphorylation and signal reception. The expression analysis of *CsCRK*s from the transcriptome data and via qRT-PCR suggested that multiple *CsCRK*s are involved in cucumber defense responses against both biotic and abiotic stresses. Overall, the study gives a basic understanding of the structural and functional attributes of the cucumber *CRK*s.

## Materials and methods

### Identification of *CRK* genes in cucumber genome and peptide property predictions

Homologous CRK sequences were searched via BLASTp on the CuGenDBv2 website (http://cucurbitgenomics.org/, accessed on 08 August 2022) by using the Arabidopsis CRK sequences. The obtained sequences were then searched for the presence of the CRK-specific domains [Stress-antifung (PF01657) and Pkinase (PF00069)] through Pfam and HMMER program with an E-value < 0.001 [36]. Afterward, the retrieved candidates were screened through the Conserved Domain Database (CDD) and Simple Modular Architecture Research Tool (SMART) tools to further confirm [37, 38]. Corresponding genomic DNA sequences, coding DNA sequences (CDS), and other related sequences were retrieved from the Cucumber genome v3 [39]. Various peptide properties, such as molecular weight, amino acid length, isoelectric point (pI), and grand average of hydropathicity (GRAVY) of the CsCRKs were predicted by the ProtParam tool [40].

### Sequence alignment, conserved motif, and phylogenetic analysis of CsCRKs

The Multiple Expectation Maximization for Motif Elicitation (MEME) tool was employed to analyze the de novo motifs in CsCRKs [41]. Multiple sequence alignments were carried out by using the CsCRKs along with *Arabidopsis thaliana* (AtCRKs) and rice (OsCRKs) in Clustal Omega [42]. A phylogenetic tree was then constructed by using the aligned sequences using the Molecular Evolutionary Genetics Analysis (MEGA v11) program with 1000 bootstraps [43].

### Gene structure, *cis*-regulatory element, and subcellular localization analysis of *CsCRK*s

The gene structure and exon-intron arrangements of *CsCRK*s were analyzed using the Gene Structure Display Server program [44]. About 2Kb upstream sequence of the *CsCRK*s was taken for the *cis*-regulatory element analysis by using the PlantCARE tool [45]. The mGOASVM server for plants was used to predict the CsCRK subcellular localizations [46]. 

### Chromosomal distribution, gene duplication, and synteny analysis

The chromosomal distribution map of the *CsCRK*s was created by using the MapGene2Chrom (MG2C) tool [47]. For the identified *CsCRK*s, the gene duplication, synteny, and collinearity analysis were performed on TBtools software [48]. The Ka/Ks Calculator 2.0 was used to deduce the homologous gene pairs for the gene duplication event analysis [49]. Similarly, the genome sequence and annotation files in FASTA and gff/gtf formats were downloaded from Cucumber genome v3 and Arabidopsis genome (TAIR) and used in the synteny analysis by using the one-step MCScanX tool in TBtools and visualized by using the dual synteny plot.

### Gene ontology and protein interaction network prediction

The gene ontology (GO) analysis was performed using the Blast2GO and WEGO2.0 tools [50]. The String program (https://string-db.org/) was used with the default parameters to analyze the protein-protein interaction network of CsCRKs and Cytoscape was used to visualize the results [51].

### Plant and pathogen materials

The cucumber variety “Barsharani” was used as the plant material for this study. The seeds were surface sterilized and grown in plastic pots (15 cm diameter) with sterile soil and manure mix in a greenhouse maintained at 28 ± 2 ^o^C and relative humidity of 80 ± 5%. A virulent strain of *S. rolfsii* was obtained originally from the ICAR-National Bureau of Agriculturally Important Microorganisms and was used as the pathogen for the study.

### Inoculation of cucumber plants with *S. rolfsii*

From a 12-day-old *S. rolfsii* culture plate, maintained on potato dextrose agar (PDA) media, the inoculum (mycelial plug) was transferred to 500 g of pre-cooked autoclaved Sorghum seeds [52]. The inoculated Sorghum seeds were kept at 28 ± 2 ^o^C for a week before next its use. Soil mixed with manure was autoclaved and then mixed with the incubated Sorghum seeds in a concentration of 10 g seeds/1 kg of soil [53]. The soil-Sorghum seed mixtures were filled into plastic pots (15 cm diameter) and kept at natural conditions in the greenhouse for 10 days. Twenty days old cucumber seedlings were transplanted into the pots containing soil-Sorghum seed mixtures to facilitate the *S. rolfsii* infection. Seedlings transplanted onto only sterilized soil-filled pots served as the control for this experiment. The experiment was performed with three independent replications.

### Isolation of total RNA and synthesis of first-strand cDNA

Cucumber leaves were collected from the plants after transplanting them in the *S. rolfsii*-infected soil on days 0, 1, 3, 5, and 7 (DPI, days post-inoculated), and immediately stored at -70 ^o^C after freezing them with liquid nitrogen. From the collected tissues, total RNA was extracted by using the TRIzol reagent (Thermo Fisher, Waltham, Massachusetts, United States). The RNA quality and quantity were checked on a QIAxpert System (Hilden, Germany) and by running a 0.8% (w/v) agarose gel. Subsequently, Then, the first-strand cDNA was synthesized by using a Verso cDNA Synthesis Kit (Thermo Fisher, Waltham, Massachusetts, United States) and stored at -70 ^o^C.

### Expression analysis of*CsCRK*s under stress conditions from cucumber transcriptome data

The expression of the identified 15 *CsCRK*s was analyzed under two stress conditions, including chilling (abiotic, keeping the cucumber plants at 4 ^o^C) and powdery mildew (PM) infection (biotic) stress. The cucumber transcriptome data obtained from the bioprojects PRJNA438923 and PRJNA321023 was used to evaluate the expression dynamics of *CsCRK*s under chilling and PM stress, respectively. The PM-responsive expression data was investigated at 2 DPI and the cold stress-responsive expression was checked at 0, 2, 6, and 12 h of stress subjection. The expressions of *CsCRK*s were visualized by constructing a heatmap using TBtools software [48].

### Expression analysis of *CsCRK* in response to*S. rolfsii*

The expression of the cucumber *CRK* genes was analyzed by performing a real-time quantitative polymerase chain reaction (qRT-PCR) on a Roche Light Cycler (Basel, Switzerland) in a total volume of 10 µL per reaction. The reaction mixture consisted of SYBR green master mix (5 µL), gene-specific primers (Table S1) (2 µL), miliQ ultrapure water (2 µL), and 10 folds diluted cDNA (1 µL). The cucumber actin gene (*CsActin*) was used as the reference gene to calculate the relative expressions via the 2^−∆∆Ct^ method (Livak and Schmittgen, 2001). Three independent biological replicates were included to perform the experiment and each biological replicate consisted of three technical replicates. The expression data was statistically verified by performing a one-way analysis of variance (ANOVA) at *P* ≤ 0.05 using the Data Processing System software.

## Supplementary Information

**Additional file 1.**

**Additional file 2.**

**Additional file 3.**

## Data Availability

All data generated or analyzed in this study are included in this published article and its supplementary material. The datasets analyzed during the current study are available in CuGenDBv2 website (http://cucurbitgenomics.org/, accessed on 08 August 2022) and in the public bioprojects PRJNA438923 and PRJNA321023 at NCBI.
