# Supplementary material for: Genome-wide identification and molecular characterization of CRK gene family in cucumber (Cucumis sativus L.) under cold stress and sclerotium rolfsii infection
Source: BMC Genomics. 2023 Apr 26;24:219. doi: 10.1186/s12864-023-09319-z (PMC10131431; doi:10.1186/s12864-023-09319-z)
Supplement: Supplementary file 1 — Additional file 1. [file 12864_2023_9319_MOESM1_ESM.docx]

**Table S1.** List of the primers used in the *CsCRK* expression analysis.

| **Gene name** | **Gene ID** | **Forward primer (5'→3′)** | **Reverse primer (5'→3′)** |
| --- | --- | --- | --- |
| *CsCRK1* | CsaV3_3G042660 | AATACTTCCATCGCCCGACC | CCTTGGCTCAGGGGACAAAA |
| *CsCRK2* | CsaV3_5G031370 | AACAGCACCTATCACGCCAA | TGGTTGCGGTGGCTAAGAAA |
| *CsCRK3* | CsaV3_2G024830 | TCAGCTACTGGGTTTTTGCATC | GAAACAGAATCAACGGGGAGA |
| *CsCRK4* | CsaV3_1G011140 | GTTTGTTGGCGTTGGCACTA | GTGAAGGTGGTGAGGAGGTG |
| *CsCRK5* | CsaV3_6G049310 | TCGCTTCATTTTTGGCACAAT | ATGCTCTTCCTCCATGGTCAG |
| *CsCRK6* | CsaV3_6G049370 | AGAGCACAACACAACTGCCT | CGAGTCCGTAGTTAGCGTCC |
| *CsCRK7* | CsaV3_6G049380 | TTCCACTCCACCGATGTTCG | CATACCGGAGGAAGCAACCA |
| *CsCRK8* | CsaV3_1G011160 | TGGGTTTGCTGTAGGATCGG | AGCCAGCATTCAACACCCTT |
| *CsCRK9* | CsaV3_6G049360 | GTGTTGTTATTGGGGACGCC | ACAGCTCCAAATCCACCCTG |
| *CsCRK10* | CsaV3_6G049320 | TTGAATTGTCTCCGCCTCCA | AAACGACGGAGACCACAACA |
| *CsCRK11* | CsaV3_6G049350 | GCAGCCGCACACTTCATATTT | CGCGTGTTGGAAGAGATGGA |
| *CsCRK12* | CsaV3_2G024820 | CCTCGTCGTCCAACACTCAA | TCGCCAGAAGTGGCATTGTA |
| *CsCRK13* | CsaV3_1G011280 | GCTACCAGATTCGCCTCCAG | ACTTGTTCACCCTGTCGTCT |
| *CsCRK14* | CsaV3_7G033220 | TGTTGTAGCCAACGTTATTTATCCT | TCGATGCAGGTACGACAAG |
| *CsCRK15* | CsaV3_1G011200 | GTAGCGGTTGTAATGGCGGA | GACAAAGATGCCAGTGGGTG |
| *CsActin* | CsaV3_4G024970 | GTCGGAATGGGGCAGAAAGA | GTTCTTCTGGAGCGACACGA |
| *CsGAPDH* | CsaV3_4G037480 | CTCAGCTTACCCCCAAGGCAG | AGCAAGTGGGAGGCATTCTTG |
