# Supplementary material for: Genome-wide identification and molecular characterization of CRK gene family in cucumber (Cucumis sativus L.) under cold stress and sclerotium rolfsii infection
Source: BMC Genomics. 2023 Apr 26;24:219. doi: 10.1186/s12864-023-09319-z (PMC10131431; doi:10.1186/s12864-023-09319-z)
Supplement: Supplementary file 2 — Additional file 2. [file 12864_2023_9319_MOESM2_ESM.docx]

**Table S2.** The Ka, Ks, and Ka/Ks values of the *CsCRK*s.

| **Gene pairs** | **Ka** | **Ks** | **Ka/Ks** |
| --- | --- | --- | --- |
| *CsCRK10-CsCRK12* | 0.098679065 | 0.20765 | 0.475218228 |
| *CsCRK11-CsCRK3* | 0.234 | 0.4144 | 0.564671815 |
| *CsCRK4-CsCRK9* | 0.1801 | 0.35685 | 0.504693849 |
| *CsCRK2-CsCRK1* | 0.091964195 | 0.1989 | 0.462363977 |
| *CsCRK14-CsCRK8* | 0.26735 | 0.42385 | 0.630765601 |
| *CsCRK5-CsCRK6* | 0.1057 | 0.2911 | 0.363105462 |
