# Supplementary figures and images for: Genome-wide identification and molecular characterization of CRK gene family in cucumber (Cucumis sativus L.) under cold stress and sclerotium rolfsii infection
Source: BMC Genomics. 2023 Apr 26;24:219. doi: 10.1186/s12864-023-09319-z (PMC10131431; doi:10.1186/s12864-023-09319-z)

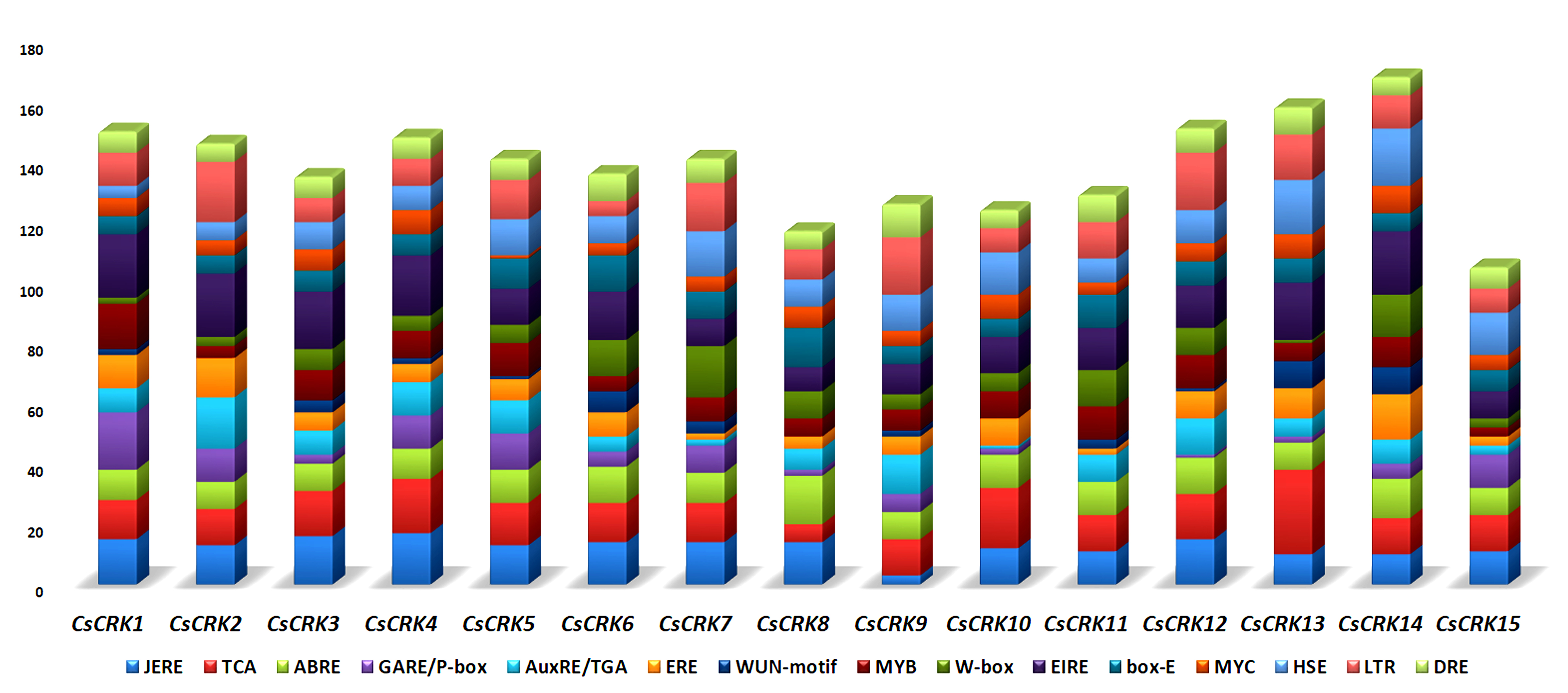

Supplement: Supplementary file 3 — Additional file 3. [file 12864_2023_9319_MOESM3_ESM.jpg]
